# Innovative Systems to Deliver Allergen Powder for Epicutaneous Immunotherapy

**DOI:** 10.3389/fimmu.2021.647954

**Published:** 2021-03-26

**Authors:** Yensheng Wang, Yifei Kong, Mei X. Wu

**Affiliations:** Wellman Center for Photomedicine, Department of Dermatology, Massachusetts General Hospital, Harvard Medical School, Boston, MA, United States

**Keywords:** epicutaneous immunotherapy, microneedles, powdered allergens, tolerance, adjuvants

## Abstract

Allergy is a disorder owing to hyperimmune responses to a particular kind of substance like food and the disease remains a serious healthcare burden worldwide. This unpleasant and sometimes fatal allergic disease has been tackled vigorously by allergen-specific immunotherapy over a century, but the progress made so far is far from satisfactory for some allergies. Herein, we introduce innovative, allergen powder-based epicutaneous immunotherapies (EPIT), which could potentially serve to generate a new stream of technological possibilities that embrace the features of super safety and efficacious immunotherapy by manipulating the plasticity of the skin immune system *via* sufficient delivery of not only allergens but also tolerogenic adjuvants. We attempt to lay a framework to help understand immune physiology of the skin, epicutaneous delivery of powdered allergy, and potentials for tolerogenic adjuvants. Preclinical and clinical data are reviewed showing that deposition of allergen powder into an array of micropores in the epidermis can confer significant advantages over intradermal or subcutaneous injection of aqueous allergens or other epicutaneous delivery systems to induce immunological responses toward tolerance at little risk of anaphylaxis. Finally, the safety, cost-effectiveness, and acceptability of these novel EPITs are discussed, which offers the perspective of future immunotherapies with all desirable features.

## Introduction

Allergic diseases have been steadily rising and approximately 50 million Americans or 20% of the population in the United States are now affected by one or more allergic conditions (1). Among these allergic conditions, 220 to 520 million people are allergic to one or more foods, which disproportionally affects children and people in the industrialized countries (2). For instance, an estimated 3.2 million Americans are allergic to peanuts, these patients are at a daily risk of peanut anaphylaxis, and yet few treatment options are available to them besides strict dietary avoidance and carrying medication at all times for immediate risk-relief like an adrenaline autoinjector (2, 3). Childhood food allergy costs an estimated $24.8 billion annually, on average of $4,184 a year per child, in which the direct medical cost is about $4.3 billion a year, including clinician visits, emergency department visits, and hospitalizations. Caregivers have reported a willingness to pay $20.8 billion a year or $3,504 a year per child for food allergy treatment alone (4). Cost-effective analysis also estimates an incremental cost-effectiveness ratio (ICER) of $2,142 per quality-adjusted life-year (QALY) when intervention was compared to simple avoidance. Allergen-specific immunotherapy (SIT) would lead to incremental improvements of 1.15 QALY while costing $2,463 more than the avoidance group over the 20-year model time horizon (4). However, the estimation model was based on oral immunotherapy that reported 12% of patients receiving epinephrine during the treatment period of allergen escalation and 6% receiving epinephrine during the maintenance, where handling the severe adverse events took a great part of the heath care spending.

Over a century, scientists have been looking for the cure to the allergic diseases (5). The first successful clinical study was dated back to 1911 when Leonard Noon and John Freeman developed a protocol of subcutaneous injections of pollen extracts with increasing doses according to a defined schedule for patients with hay fever (6). The allergen-SIT resulted in hyposensitization that was significantly more effectively induced in a higher dose than in a lower dose of pollen allergens for treating hay fever (6). This concept has been since implemented in treatment of all allergies (5, 7). However, due to a high risk of anaphylaxis, a long period of treatment required, and a low therapeutic efficacy, SIT is only practiced in the clinics for some allergies and new therapeutic concepts have continuously emerged for more effectively and safely tackling other allergies like peanut allergy. Most of the current therapeutic approaches are using chemical allergoids, oral immunotherapy (OIT), sublingual immunotherapy (SLIT), subcutaneous immunotherapy (SCIT), and epicutaneous immunotherapy (EPIT) with or without concurrent biological immune modifiers such as Omalizumab, an anti-IgE antibody (2). Other experimental methods in development are DNA vaccine and gene therapy (5, 8). Yet, all these SITs are moderately effective and require more than 50 treatments over 2~3 years to have temporarily effects, so that only <5% of patients choose these treatments.

## The Skin Is a Safe and Effective Site for Immunotherapy

**Figure 1:**
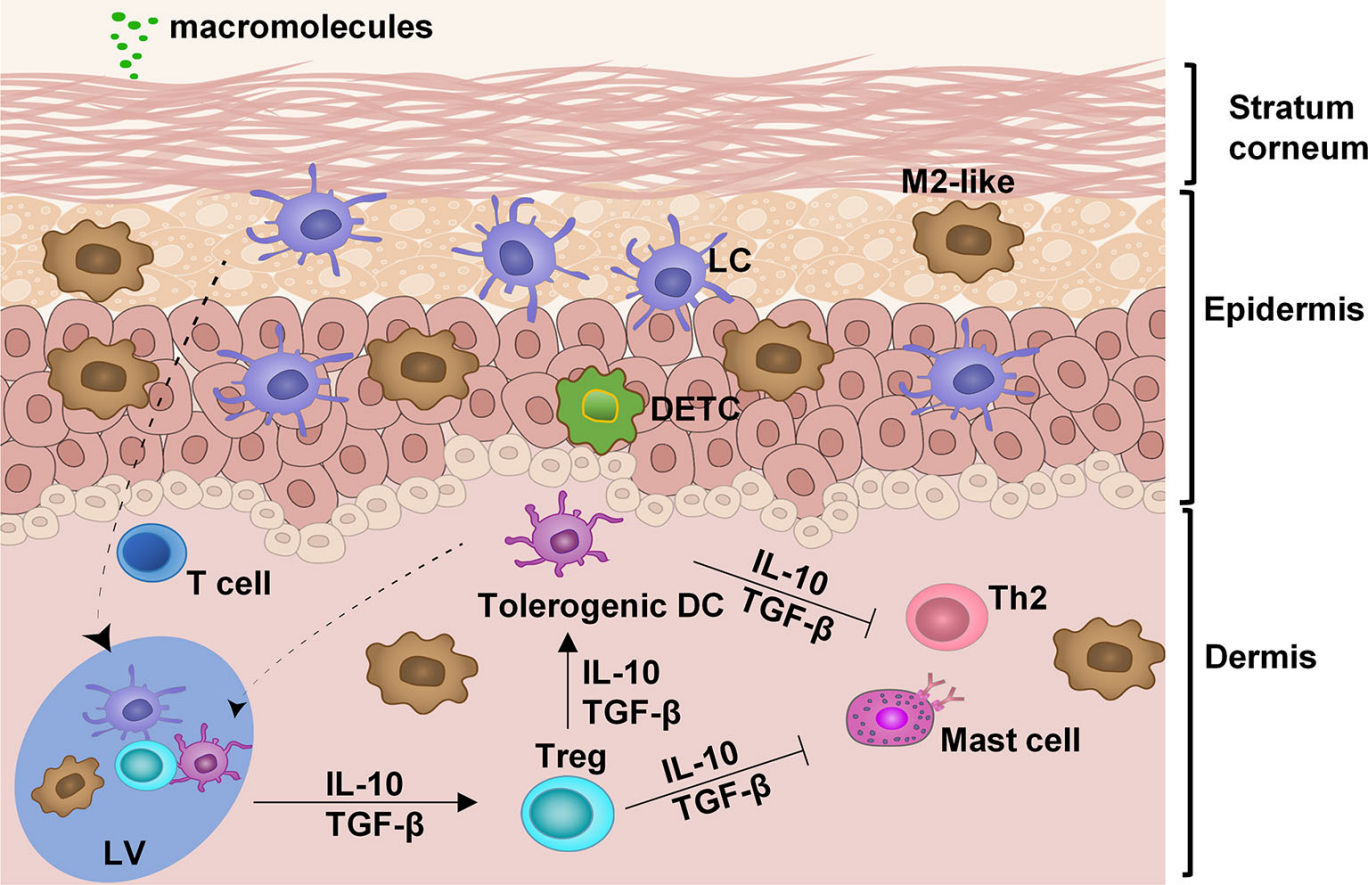
Anatomy and cell composition of the skin. In the absence of any insult, the skin is retained at a steady state by interplays among different immune cells: LC, Langerhans cells; M2-like, M2-like tissue resident macrophages; DETC, dendritic epidermal T cells; tolerogenic dendritic cells (DC); Treg, T regulatory cells; and skin-resident T cells. These immune regulatory cells work in concert to suppress the hyperimmune reaction of type 2 help T cells (Th2) and mast cells. LV, lymphatic vessel.

Skin is the biggest organ system in our body and constantly encounters massive environmental insults due to its large surface area. It must rigorously keep a balance between defending hazardous pathogens and preventing overreaction to the innocuous substances. The stratum corneum, the outermost layer, of the skin comprises layers of specialized skin cells, also called horny layer and serves as a physical barrier to separate external from internal insults (**Figure 1**). It is impermeable to macromolecules and thus delivery of allergens, most of which are large in sizes, through intact skin, is extremely challenging (**Figure 1**). The epidermis beneath the stratum corneum is an epithelial layer primarily composed of keratinocytes, Langerhans cells (LCs), macrophages, and dendritic epidermal T cells (DETCs). In a steady state, most LCs are restricted to the epidermis and only a small fraction, about 2–3%, are mobile and constantly moving from the skin to the draining lymph nodes (DLN) *via* the lymphatic vessels in the dermis to present self-antigens and establish the immune tolerance in homeostatic conditions (9). The epithelial cells are able to divide rapidly around a wound once it occurs, migrate across the wound and close it, making it possible for a micropore at a size of 10-times smaller than a hair to be sealed within 2-4 hours to restore the skin barrier function and fully closed within 15-40 hours as unraveled by a clinical study of micropore closure kinetics (10–13). This fast sealing characteristic is essential for the first-line body defense and epidermal barrier integrity and has been well appreciated in skin resurfacing (13–16). This unique feature of the skin raises an intriguing possibility that allergens can be sufficiently delivered into the epidermis *via* an array of micropores without incurring any overt irritation of the skin. Apart from fast healing, the epidermis is *a non-vascularized tissue* that limits an entrance of allergens into the bloodstream and averts anaphylaxis. The dermis is a stromal layer immediately below the epidermis wherein a variety of immune cells can be found, including T cells, mast cells, macrophages, and dendritic cells (DCs) (17, 18).

The skin is long recognized as a preferable site for tolerance induction. The complex interplay among various immune cells maintains skin homeostasis. In the absence of local inflammation, skin DCs remain immature with a low surface expression of MHC class II and costimulatory molecules, reflecting their participation in the maintenance of peripheral immune tolerance by induction of T regulatory (Treg) cells and T‐cell anergy/deletion (19–22). Treg cells are generated in the draining lymph nodes and circulated back to tissues where allergens are found. In the tissues, Treg cells constantly guide DCs to retain a tolerogenic state by secreting tolerogenic cytokines IL-10 and TGF-β (**Figure 1**) (19). Treg cells also suppress mast cells and Th2 cells rendering them unresponsive to allergens. M2-like tissue-resident macrophages are another major subset of tissue-resident macrophages and exhibit immunoregulatory and hypo-stimulatory properties that are sustained after migration to the secondary lymphoid organs to induce antigen-specific Tregs (23, 24). These anti-inflammatory M2-macrophages are essential effector cells in mediating hypo-responsiveness following EPIT (25). In addition, they also play an essential role in scavenging degraded intermediates of self-macromolecules to maintain the immunotolerant environment of the skin (17, 26). Cell to cell cooperation in orchestrating tolerogenic responses is the cornerstone in maintaining skin homeostasis. The balance in inflammatory responses or tolerance responses is a complex system that recent studies have been scrutinized (9, 17, 27, 28).

Emerging findings indicate that skin-derived tolerance has a unique property of systemic effects. It has been shown that EPIT exerts tolerogenic effects that are not limited to local desensitization and can be extended to the gut mitigating food allergy or the airway alleviating hyperresponsiveness to allergens in the respiratory system (29–31). Moreover, recent studies unraveled that skin-derived T cells and blood-derived T cells expressed a different set of genes involved in tissue homing and cell activation (32, 33). Treg cells induced *via* skin immunization express the characteristic regulators in guiding the migration toward respiratory and gastrointestinal systems in addition to the skin. These regulators include cutaneous lymphocyte antigen (CLA) and chemokine receptors CCR3, CCR4, CCR6, CXCR3, CCR8, and CCR9 (34, 35). In accordance with this, EPIT proved efficacious in alleviation of bronchial hyper-responsiveness, eosinophil recruitment in the skin, and food allergy (29, 30, 36). The finding that skin-derived tolerance manifests a global effect rather than local desensitization opens a window to the immunological engineering that could modulate the systemic tolerance and destination-targeting signaling *via* the skin.

## Conventional “Epicutaneous” Immunotherapy

EPIT was initiated over a century ago but it has been successful in treatment of only some allergies. One of the major challenges for EPIT is how to deliver a sufficient amount of allergens into the epidermis through intact skin without incurring too much Th2 immune response because a majority of allergens are macromolecules and cannot penetrate through the stratum corneum. To circumvent this barrier, Vallery-Radot prepared the skin for immunotherapy by scarification, followed with an allergen applied onto the scarified skin dated back to 1921. Dropping allergen extract onto scarified skin or rubbed skin alleviated allergic symptom in a number of studies (37, 38). These pioneer EPITs however did not fully realize the advantage of the skin’s innate immune properties, but rather utilizing scarified skin mainly to bypass the stratum corneum barrier to deliver allergen to the epidermis, which however induces unwanted Th2 immune responses (38). In many cases, skin scarification itself can worsen allergic responses, because the skin is sensitive to various insults and invaders and can be a site for inducing either sensitization or immune tolerance. To mitigate these adverse events, gentle physical disruption of the skin by tape-stripping was attempted in place of scarification in humans. Although tape-stripping significantly increased penetration of allergens into epidermis (39), this physical skin aberration, similar to scarification, also provoked release of pro-inflammatory cytokines like thymic stromal lymphopoietin (TSLP), T_H_2 immune responses, and allergic sensitization (40).

Alternatively, intradermal (ID) administration was investigated to minimize skin damage, but it required skillful medical workers to use the Mantoux technique. There is no guarantee to be successful for every injection. In case allergens were administered into an inappropriate depth, it could cause anaphylaxis. ID injection has been recently improved with a small, thin, 1.15 mm long needle pressing perpendicularly to the skin, which injects a very small volume (2 µl) (36G ID injection system from Terumo). The small and thin needle warrants not only intradermal delivery but also no need for skilled healthcare workers to do the injection (41). In comparison with dropping allergen solution directly onto tap-stripped skin, ID-mediated EPIT significantly diminished allergen-specific IgE production while increasing IgG production in sensitized mice (41). Although ID-EPIT is safer than SCIT, it is disappointing for its low efficacy compared with SCIT, OIT or SLIT, largely because a limited volume can be inoculated into the skin. On the other hand, a large volume administered comes with high levels of skin reactogenicity. A growing body of evidence suggests that allergen activates antigen-presenting cells (APCs) in the epidermis, promotes allergen-specific Treg cells, and significantly inhibits allergic responses, which occurs best in intact skin (35, 36). Any significant damage of the Skin can breach the skin barrier causing type 2 immune response that can worsen IgE-mediated allergic responses.

## Innovative Epidermal Powder Delivery Systems

### Viaskin

To minimize type 2 immune responses of the skin, Viaskin is designed to facilitate diffusion of powdered allergen from skin surface to the epidermis through intact skin (42). It is engineered by electronically spreading powdered allergens onto a supporting membrane that is sealed in a chamber. When applied on the skin, Viaskin creates an occlusive chamber on the skin in which moisture is rapidly generated and accumulated, solubilizing the allergens in the supporting membrane. The powdered allergens are gradually solubilized and slowly released from the supporting membrane, allowing it to penetrate the epidermis *via* the skin surface (36, 43). The delivery system doesn’t damage the skin or cause significant Th2 immune response. Clinical studies showed that Viaskin provoked less than 20% mild nonpatch‐site reactions with the treatment success of 45.8% in 100 µg group and 48% in 250 µg group (p=.003 and p=.005, respectively) as compared to 12% in the placebo group in a phase IIb trial (42, 44). In phase III trials, EPIT using 250 µg Viaskin significantly improved the allergy symptom by 35.5% in children aged 4-11 years after 12 months of treatment compared to 13.6% in the placebo group (p<0.001: 95% confidence interval = 12.4-29.8%) (42, 45). While successfully increasing peanut tolerance, Viaskin-mediated EPIT did not evoke anaphylaxis in the clinical study, reaffirming super safety of the EPIT. However, the treatment was not effective in patients at age >11 years who may have thicker and drier skin than younger ones, moisture of which may not be sufficient for allergen penetration. It is also possible that an allergen dose delivered by a Viaskin diminishes in proportion to an increase of body weight and thus the allergen dose as µg/kg is considerably lower once toddlers grow up.

Viaskin has recently received fast track and breakthrough therapy designation from the U.S. Food and Drug Administration (FDA) for the treatment of peanut allergy in children ages 4 to 11. Although Viaskin-mediated EPIT has a better safety effect, its efficacy is modest and the treatment benefits only a subgroup of patients (42, 44–46). This limitation is ascribed primarily to its insufficient delivery of allergens into the skin. Viaskin delivers only less than 10% allergen in the supporting membrane into the epidermis after a 24-hr application, whereas prolonged patch wearing causes significant skin irritation (47–50). Moreover, Viaskin is limited to deliver water soluble allergens only and it would be also challenging to add tolerogenic adjuvants to the system.

### Ablative Fractional Laser for More Sufficient Epidermal Delivery

It has been known for a long time that dosage pertains to the level of tolerance; more, higher intensity of treatment fosters a greater probability of tolerance, as demonstrated by a number of studies regardless of whether OIT, SLIT, or EPIT are employed (31, 44, 46, 51). However, a high allergen dose is more likely associated with untoward adverse events, particularly life-threatening anaphylaxis, which remains the major concern. To increase the delivery efficacy without provoking untoward adverse events, ablative fractional laser (AFL) was attempted to generate a microchannel array in the epidermis followed by topical application of a powder allergen-coated array patch (52–55). The powdered allergens delivered within the microchannels are hydrated by interstitial fluid drawn into the microchannels, gradually dissolving and spreading over the epidermis. A majority (80%) of the allergens on the patch could be delivered into the epidermis in 1 hr *in vivo* in mouse models and ex human and pig skins (53, 54). Tolerogenic adjuvant could be readily added to the delivery system, greatly enhancing the therapeutic efficacy in the preclinical studies (53, 54).

**Figure 2:**
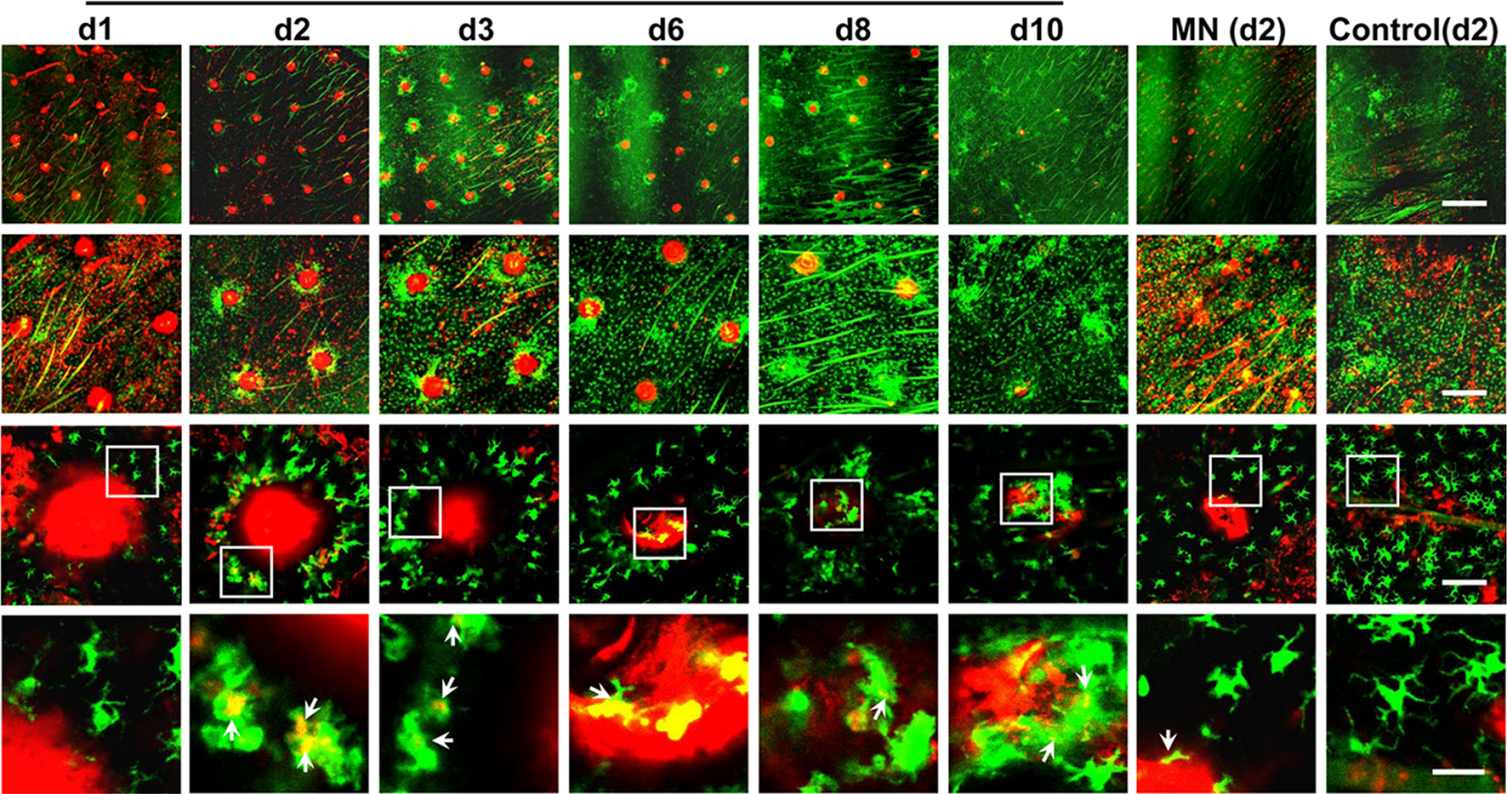
Dynamic accumulation of APCs around each powder allergen zone. Ears of MHC II-EGFP (green) mice were treated with AFL or left untreated (control) followed by topical application of ovalbumin (OVA)-coated gauze patch for 30 min. The OVA was conjugated with red fluorescence Alexa Fluor™ 647 (AF647-OVA). The epidermal layer was subjected to intravital confocal imaging at the indicated times. Representative low (1^st^ row, scale: 750µm), middle (2^nd^ row, scale: 300µm), high magnification images (3^rd^ row, scale: 75µm) are shown. Areas within the white rectangle (3^rd^ row) are enlarged to show the antigen-uptake by individual APCs (arrow, 4^th^ row, scale: 25µm). Yellow color suggests antigen-uptake by APCs. No antigen uptake occurred in untreated control ear and thus day 2 images are arbitrarily shown.

Remarkably, after the powdered allergen patch was applied onto laser-microporated skin, a large number of APCs were attracted and accumulated gradually around each microchannel, as captured by intravital confocal microscopy in mice expressing GFP-infused to MHC class II molecule. As can be seen in **Figure 2**, fluorescently labeled ovalbumin (OVA) powder (red) is deposited into an array of well-separated microchannels generated by AFL in the epidermis on day 1 (d1). GFP^+^ APCs migrate toward individual microchannel (red) composed of powdered OVA over time, becoming highly significant on day 2 (d2), peaking on day 3 (d3), and declining over 6 to 10 days until all powder is ingested (**Figure 2**). The skin becomes normalized at a cellular level after 10 days of patch application (**Figure 2**). On high magnification, antigen-uptake is evidenced by emerged yellow colors of green (APCs) and red (OVA) (3^rd^ and 4^th^ rows, **Figure 2**). Conceivably, allergens within each microchannel can continuously stimulate the immune system for a week, mimicking multiple doses of immunizations, which is known to favorably induce immune tolerance (54). The compartmentalized antigen-uptake and APC accumulation not only warrant efficiency of the immunotherapy, but also minimize leakage of allergens into the circulating system (54). Likewise, Korotchenko et al. applied house dust mite (HDM) into micropores generated in the skin of sensitized mice with a laser device called P.L.E.A.S.E.^®^ (Precise Laser Epidermal System from Pantec Biosolutions AG) (56). The epicutaneous laser microporation preferentially induced Treg cells over SCIT (57). The same laser-facilitated EPIT was also investigated in a mouse model of pollen allergy (58). In the study, the major birch pollen allergen Bet v 1 was neoglycoconjugated to mannan *via* mild periodate oxidation. Delivery of this DC-targeted allergens into the epidermis by laser-microporation was superior to intradermal injection in the induction of desensitization (59). However, inconvenience, safety, and cost that come with laser-microporation in the therapy remain to be resolved before it can be broadly practiced in clinics, especially for home uses.

### Microneedle Arrays (MNA)

**Figure 3:**
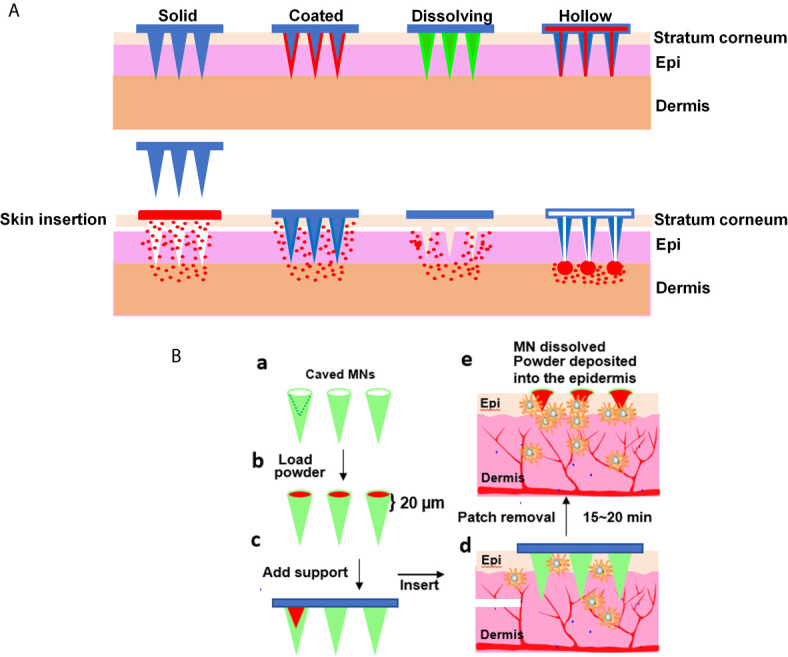
Various microneedle arrays. **(A)** Different types of microneedle arrays. From the left to right are solid, coated, dissolving, and hollow microneedle arrays. Solid microneedles are used to poke tiny holes in the skin and then removed, followed by placing an allergen-soaked patch on the pre-treated skin. Coated microneedles are inserted and remain in the skin for a while to allow coated allergens (red) dissolving off the microneedles. Dissolving microneedles are inserted into the skin and degraded gradually releasing the embedded allergens. Hollow microneedles are filled with allergen solution and deposit the allergen in the epidermis by pressure. **(B)** PLD-MNA. Green represents caved microneedles with a cave outlined in one microneedle (a). Powdered allergen (red) is loaded into the caves (b). A support (blue) is added to seal the caves and secure the array (c). After inserting into the skin for 15-20 min, the shaft of the microneedles degrades, exposing the powder in the epidermis (d, e). The powdered allergen attracts a large number of APCs around the powdered allergen (e). Epi, epidermis and 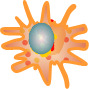, APC.

In the past decades, various types of microneedles have been developed and evaluated for transdermal drug delivery, including solid, coated, hollow, and dissolving microneedles (11, 48, 60). These microneedle patches can perpendicularly penetrate into the epidermis layer of the skin in a minimally invasive fashion. Upon microneedle application, micropores are created across the stratum corneum layer, through which any macromolecules can enter the epidermis freely. The size of a microneedle is smaller than a hair and varies from 50 to 250 µm in a length of 150–1500 µm and tip thickness of 1–25 µm. The skin micropore can be sealed in 24 hr without incurring any significant downside of the skin as described above. Solid microneedles are employed to microporate the skin resembling ablative fractional laser. Allergen-immersed patch is applied topically onto the microporated skin after removal of the microneedle array (**Figure 3A**, 1^st^ panel). The allergens on the patch enter the microchannels by the capillaries and passive diffusion into skin layers *via* the micropores. Coated microneedles come next by coating the allergen solution or allergen dispersion layer on the surface of each microneedle in the array (**Figure 3A**, 2^nd^ panel). Subsequent dissolution of allergens from the layer takes place and the allergens are delivered quickly after applying the array onto the skin. Unlike solid microneedles, dissolving microneedles are fabricated with biodegradable polymers (**Figure 3A**, 3^rd^ panel). Prior to polymerization, the drug or allergens are mixed with the mono-polymer so that the allergens or drugs can be uniformly embedded within the microneedles. Upon inserting into the skin, microneedles degrade releasing the allergens in the epidermis. The polymer can be manipulated to control a degradation rate of the microneedles and thus the rate of allergens release. The bio-acceptability and dissolution of the polymer inside the skin make it possible for releasing the allergens at a desirable pace. Among these microneedle arrays (MNAs), coated and dissolving MNAs have been investigated to deliver allergens or influenza vaccines through the skin to activate immune system (61–65). For instance, Spina et al. used microneedle arrays superficially coated with birch pollen on each microneedle to deliver the allergens into the skin in humans demonstrating an improved desensitization efficacy compared with tape-stripping or skin prick testing (39). Microneedles coated with peanut protein extract were fabricated to treat peanut allergy in murine models as well (61).

One of major drawbacks in association with the coated and dissolvable microneedles is a severe loss of antigenicity or allergenicity during microneedle fabrication. It was found that more than 50% immunogenicity lost even with additives because a repeated process of dipping and drying of the allergen was involved in the coating process. Likewise, dissolving MNAs are made of a mixture of mono-polymer and allergens followed by polymerization that could compromise the immunogenicity considerably. Hence, various excipients, stabilizers, and pH buffers must be tested to optimize the coating and polymerization procedure so that allergenicity can be well preserved. The optimization procedure is not only time-consuming but also allergen specific. For food allergens comprising multiple active ingredients, the optimization procedure remains significant hurdles as it is almost impossible to find a single recipe to preserve all active allergens sufficiently. Moreover, some of the allergens are still undefined, and the resistance of the allergens to the polymerization and quality of the allergens in the microneedles cannot be readily measured. To tackle this issue, hollow microneedles with a tiny hole through each microneedle are fabricated and filled with soluble allergens and/or adjuvants (**Figure 3A**, 4^th^ panel). Following insertion, the allergens and adjuvants can be directly pressed into the epidermis. The flow rate and release pressure can be adjusted to safely administer allergens and adjuvants without any concerns about a loss of their allergenicity or adjuvanticity.

### Powder-Laden Dissolvable Microneedle Arrays (PLD-MNA)

A new technology of a powder-laden, dissolvable microneedle array (PLD-MNA) has recently been engineered to untangle many obstacles of the aforementioned powder allergen deliveries. As depicted in **Figure 3B**, PLD-MNA is made of highly biocompatible and dissolvable hyaluronic acid (HA) or other equivalent materials with a cave in the basal of each microneedle in the array (a). The first microneedle in (a) is outlined in dash lines to show the depth and size of a cave relative to the microneedle. Each cave can be filled directly with lyophilized allergens without any modification or reconstitution, with which the immunogenicity of the allergens is 100% preserved (b). A supporting layer is added to seal the caves as well as to support the MNA (c). The shaft of caved MNA can be dissolved in 15~20 min after skin insertion (d), depositing the powder in the epidermis and attracting a large numbers of APCs (e), similar to what is seen in **Figure 2** (25, 66). The powdered allergens are retained within the epidermis for a prolonged period of time, creating an “antigen (Ag)-depot” effect. Moreover, in contrast to aqueous allergens spreading quickly into the circulation, the powdered allergens are secured in the epidermis with minimal leakage to the circulating system (25, 54).

We have demonstrated a delivery rate of 80% in 1 hr of patch application *in vivo* in mouse models (25, 66). In the preclinical study, PLD-MNA was packaged with a mixture of powdered peanut allergen (PNA), 1,25-dihydroxyvitamin D3 (VD3), and CpG. The PNA/VD3/CpG-laden MNA was more effective in treatment of peanut allergy in a murine model compared with intradermal injection (25). Powdered allergens delivered by PLD-MNA preferentially attracted immunoregulatory macrophages and stimulated the cells to produce IL-10 and TGF-β at the immunization site, resulting in an increasing number of Treg cells in lymph tissues in association with systemic tolerance. PNA/VD3/CpG-laden PLD-MNA was safer than EPIT administered intradermally or subcutaneously and reduced the number of treatments by half and the total amount of PNA and adjuvant by 80% to achieve similar outcomes as conventional ID-EPIT (25). While Viaskin’s efficacy is dependent on age working poorly in patients at age > 11 years, we don’t think this age-dependent effect is an issue for PLD-MNA as it delivers powdered allergens into the epidermis *via* micropores generated mechanically by microneedles. In addition, PLD-MNA is expected to have a shorter application time which can reduce skin reactogenicity and broaden its application at all ages. Furthermore, the ability of delivering allergens mixed with tolerogenic adjuvants in the therapy should greatly diminish the number and length of treatments, which would result in more patient complicance (25). Nevertheless, all these advantages in association with PLD-MNA-mediated EPIT wait to be corroborated in humans.

The advantages of PLD-MNA are apparent. It can deliver any allergens as long as their powder forms are available even if the molecules in the allergens are not identified. It is also accessible and could be widely implemented in clinics or home once proven in human studies. It is worthwhile to point out that a complete insertion of the PLD-MNA into the skin is not always necessary for sufficient delivery of the encapsulated powder, because the powder can be drained into the skin by the interstitial fluid influx even if the powder is placed on top of the skin. In support, we have recently shown that powder placed on top of a skin microchannel could sufficiently enter the skin *via* the microchannel as a result of the powder allergen capable of sucking interstitial fluid (67, 68). The capability of powder being drained into the skin by the interstitial fluid warrants consistency of the therapy even when the PLD-MNA be inserted imperfectly, which can happen during self-application at home. Moreover, PLD-MNA would allow a delivery of a high amount of allergen into the skin in hours with slight modification, for instance, by raising the height of the basal cave above the skin as we recently described (67). The loading capacity can be also escalated by enlarging and prolonging the microneedles for human uses owing to much thicker human skin than mouse skin and/or increasing the density of microneedles. Furthermore, with a small volume, PLD-MNA also features the convenience of storage and transportation.

## Advantage of Powder Over Aqueous Allergens for Epit

Currently, powdered allergens can be delivered into the epidermis with three technologies: Viaskin, laser-based microporation, and PLD-MNA. There are various lyophilized extracts of allergens available for skin prick testing and SIT. Those extracts can be directly loaded into PLD-MNA or microporated skin for EPIT without the need for additives, stabilizers, or excipients. Identification of the specific allergens is neither needed. Apart from allergen preservation, the powdered form of allergens can avoid chemical modification and degradation even after a long storage period compared to aqueous forms. As for PLD-MNA, the patches can be mailed to patients for home-uses and stored for a long time. No reconstitution of the allergens is required for the immunotherapy at home. Powder allergens are gradually dissolved by interstitial fluid *in situ*, which not only intrinsically creates antigen-“depot” effects, but also reduces the risk of anaphylaxis, a main concern in treating many allergies, especially food allergy. This prolonged duration of allergen release followed with PLD-MNA could constantly stimulate the immune system, mimicking daily desensitization treatment; thus, skewing the immunological responses to the tolerogenic state. On the contrary, aqueous forms of allergens administered intradermally or subcutaneously or with hollow microneedles diffused out from injection site quickly as evidenced by their increasing appearance in the circulation in a few hours after injection (25, 54). The quick diffusion increases the risk of anaphylaxis while reducing the immunotherapeutic efficacy.

Immunologically, allergens deposited by PLD-MNA attract migratory macrophages or tissue-resident macrophages leading to their accumulation around each allergen spot until all the allergen is eaten up in a manner similar to powered allergen delivered by laser-microporation described in **Figure 2** (25). The macrophages expressed IL-10 and TGF-β and migrated to the draining lymph nodes stimulating CD25^+^Foxp3^+^ Treg cells. These Treg cells could be found in the draining lymph nodes, spleen, and mesenteric lymph nodes (MLN) of allergen-sensitized mice and are associated with systemic tolerance. Under similar conditions, allergens administered by intradermal injection was significantly inferior in terms of macrophage accumulation, IL-10 and TGF-β generation, and Treg cell induction (25). Moreover, intradermal injection of allergens caused significant skin irritation and required 5-fold more peanut allergen and VD3 and CpG adjuvant for similar desensitization outcomes as compared with PLD-MNA-mediated EPIT (25). Different from PLD-MNA, allergen delivered by Viaskin was mainly captured by LCs and CD11b^+^ dermal DCs and depletion of LCs caused dramatic decreases in the efficacy of desensitization (35, 36). By capturing in the epidermis, rather than in the dermis, allergen delivered by either Viaskin or PLD-MNA effectively avoids sensitization by activated keratinocytes or APCs in the dermis. Moreover, the two EPIT stimulated the generation of Treg cells, which directly suppressed mast cell activation, leading to sustained clinical protection against food-induced anaphylaxis. Interestingly, in spite of both inducing Treg cells, Viaskin brought about more LAP^+^ Treg cells in the MLN, while PLD-MNA induced a significant number of conventional CD25^+^Foxp3^+^ Treg cells in the MLN (25, 29). These observations suggest distinct immune properties between the two EPITs although both technologies deliver powdered allergens into the epidermis. A further investigation of the underlying immune differences between the two EPIT would help us to better understand the potential of EPIT in general.

## Adjuvants for Immunotolerant Propensity

Only three adjuvants have been licensed by FDA for human vaccines so far: i.e. Alum, monophosphoryl lipid A (MPL), a TLR4 agonist, and MF59, but all three are approved for boosting vaccines not for allergen-specific immunotherapy. Similar to adjuvant in vaccines that can bolster the vaccine efficacy, adjuvants can also amplify tolerant immune responses that are expected to substantially improve allergen-specific immunotherapy. These adjuvants are also called tolerogenic adjuvants. Skin-derived immunotherapy with adjuvant has been proposed to modify the cytokine environment and direct the immunological response toward a tolerogenic state. Several studies have shown adjuvant application could enhance tolerance in treating allergy (54, 67–69). To date, tolerogenic adjuvants remain largely under investigated. Most of tolerogenic adjuvants are defined or screened initially by their ability to suppress immune responses elicited by a vaccine in non-sensitized subjects, which are inappropriate as immune suppressive effects vary substantially in sensitized vs. non-sensitized individuals. Another type of adjuvant for tolerance induction that is commonly tested is the adjuvant that promotes Th1 immune responses. These two types of adjuvants may not be sufficient. Tolerogenic adjuvants should be more extensively investigated in allergen-sensitized subjects as these subjects respond to a given adjuvant very differently from those non-sensitized subjects.

We screened various prominent experimental adjuvants for their ability to induce anti-inflammatory cytokines like IL-10 and TGF-β at the site of ID immunization because of an importance of the cytokines in the induction of Treg cells (54). We found that a combination of VD3 and CpG could be a competent tolerogenic adjuvant not only because they had a safety profile but also because they appeared to have the best tolerogenic effect among a group of prominent experimental adjuvants tested (54). In the preclinical study, the pair displays more effective in alleviating allergic responses, comparing to CpG alone or CpG + rapamycin (54). VD3 can be speculated to be a great adjuvant candidate because in the skin, tolerogenic function of DCs is influenced by VD3 (70, 71). An e*x-vivo* study has suggested treatment of DCs with VD3 could elicit Treg-inducing tolerogenic DCs (72). Exposure to VD3 can inhibit the expression of MHC class II, CD80, and CD86 on DCs with a high ratio of PD-L1/CD86, while reducing the production of pro-inflammatory cytokines such as IL-12 and IL-23, and increasing TGF- β and IL-10 production. Although retinoid acid also plays a role in triggering tolerance, retinoid acid (RA) appears not to be the best candidate in epicutaneous immunotherapy in the basis of our observation (54). It is because there are much fewer RA-producing DCs in skin-draining lymph nodes than in the intestinal tract (73). VD3 favored Treg cell development and blocked B-cell proliferation and differentiation toward antibody-producing plasma cells; it is therefore a potential adjuvant candidate in epicutaneous immunotherapy.

CpG, a TLR9 agonist, is also indicated as a potential adjuvant for EPIT. Previous studies suggested that epicutaneous immunization with OVA and CpG reduced the production of OVA-specific IgE and Th2 cytokines including IL-4, IL-5 and IL-13, concomitant with increased synthesis of OVA-specific IgG2a antibodies (54, 69). In a double-blind, placebo-controlled, phase II clinical trial, subcutaneous injection of ragweed pollen antigen conjugated to CpG motif demonstrated suppression of antigen-specific IgE antibody (74). Immunomodulation by CpG has been found to prevent allergic symptoms in experimental animal models as well (25, 54). Our recent observations suggested that stimulation of IL10 and TGF-β in skin resident macrophages by VD3 and CpG could lead to enhanced induction of Treg cells (25). Even though epicutaneous immunotherapy is already demonstrated to be safe and effective, adding adjuvants could create a tolerogenic microenvironment that sustains allergenic tolerance and serves as a safer strategy in controlling the untoward anaphylaxis.

Various anti‐inflammatory cytokines and immunosuppressive agents can program DCs to acquire tolerogenic properties and promote the induction of IL‐10, Indoleamine 2, 3-dioxygenase (IDO), and TGF‐β that are critical for promoting Treg cell responses or inducing the expression of cell surface molecules such as ILT3/4, PDL1/2, ICOS‐L, B7.H, CD95L, which promote T‐cell anergy or deletion or Treg cells (19, 75, 76). These studies emphasize the major role to play with cellular interactions and the microenvironment in programming tolerogenic DCs and macrophages, forming a basis for initial screening novel tolerogenic adjuvants. Further understanding how various suppressive cytokines and surface molecules govern the central and peripheral tolerance is essential for identifying novel adjuvants for effective and sustained SIT.

## Discussion

Powder allergen-based immunotherapy represents a future trend of EPIT. PLD-MNA, Viaskin, and laser-mediated microporation can sufficiently carry powdered allergens into epidermis with minimal skin reaction. These innovative delivery technologies are able to fully preserve the allergenicity and/or adjuvant, programming tolerogenic microenvironment that rewires the immunological response to induce tolerance. PLD-MNA is ready-to-test for clinical trials in treatment of miscellaneous allergies, should PLD-MNA be fabricated in a large-scale Good Manufacturing Practice (GMP). In comparison with Vaskin-mediated EPIT that relies on the permeability of a specific allergen into the epidermis *via* moisture and intact skin, PLD-MNA has a much higher powder delivery rate and displays a feature of sustained release as well as prolonged stimulation of the immune system if it can be proven in humans. Future investigation should further unravel the intertwined mechanism of skin-resident tolerogenic APCs, especially tolerogenic macrophages and Treg cells and underneath immunological signaling as these modulation programs will delineate a future immunological manipulation that controls the tolerogenic or immunogenic immune responses in vulnerable population.

## Author Contributions 

All three authors participate in initial discussion and composition of the review. YW wrote the first draft. YK revised it and added some content, and MW finalized the review. All authors contributed to the article and approved the submitted version.

## Funding

This work is supported by National Institute of Health grants AI135233 and AI149012 and the Wellman Center Discretionary Fund (to MW).

## Conflict of Interest

The authors declare that the research was conducted in the absence of any commercial or financial relationships that could be construed as a potential conflict of interest.

## Abbreviations

AFL, Ablative fractional lasers; APCs, Antigen-presenting cells; B7.H. B7 costimulatory ligand homolog; BCG, Bacillus Calmette–Guérin; CCR, C–C chemokine receptor; CD, Cluster of Differentiation; CLA, Cutaneous lymphocyte antigen; CpG, Cytosine-phosphate-guanine; CXCR, C–X–C chemokine receptor; DCs, Dendritic cells; DETC, Dendritic epidermal T cells; EPIT, Epicutaneous immunotherapy; GFP, Green fluorescent protein; GMP, Good Manufacturing Practice; HA, Hyaluronic acid; ICER, Incremental cost-effectiveness ratio; ICOS-L, Inducible co-stimulatory molecule ligand; IDO, Indoleamine 2, 3-dioxygenase; IgE, Immunoglobulin E; IL, Interleukin; ILT, Inhibitory Ig-like transcripts; LCs, Langerhans cells; MHC, Major histocompatibility complex; MNA, Microneedle array; MPL, Monophosphoryl lipid; OIT, Oral immunotherapy; OVA, Ovalbumin; PDL, Programmed cell death ligand; PLD-MNA, Powder-laden, dissolvable microneedle array; PRR, Pattern recognition receptor; QALY, Quality-adjusted life year; RR, Relative risk; SCIT, Subcutaneous immunotherapy; SLIT, Sublingual immunotherapy; TGF-β, Transforming growth factor beta; TLR, Toll-Like Receptor; Treg, Regulatory T cells; VD3, Vitamin D3 or 1,25-dihydroxyvitamin D3.
